# Cervical cancer screening programs and their context-dependent effect on inequalities in screening uptake: a dynamic interplay between public health policy and welfare state redistribution

**DOI:** 10.1186/s12939-021-01548-6

**Published:** 2021-09-24

**Authors:** Vincent De Prez, Vladimir Jolidon, Barbara Willems, Stéphane Cullati, Claudine Burton-Jeangros, Piet Bracke

**Affiliations:** 1 Department of Sociology, Ghent University, Korte Meer 5, 9000 Ghent, Belgium; 2 Institute of Sociological Research, Geneva School of Social Sciences, University of Geneva, 40 bd du Pont-d’Arve, CH- 1211 Geneve 4, Switzerland; 3 Population Health Laboratory, Department of Community Health, Faculty of Science and Medicine, University of Fribourg, Ch. du Musée 8, CH-1700 Fribourg, Switzerland

**Keywords:** Cancer screening participation, Education gradient, Income gradient, Screening strategy, Access to healthcare, Decommodification

## Abstract

**Background:**

While organized and opportunistic cervical cancer screening (CCS) programs implemented across the European Union have increased participation rates, barriers to socioeconomically deprived women remain substantial, implying high levels of inequality in CCS uptake.

**Aim:**

This study assesses how the *screening strategy* (as a score based on the availability of organized population-based CCS programs), *accessibility of the healthcare system* (as an index of out-of-pocket expenditure as a proportion of total healthcare costs, public health expenditure as a percentage of total GDP, and general practitioner (GP) density per 10′000 inhabitants) and *social protection* (as a decommodification index), impact education- and income-based inequalities in CCS uptake.

**Methods:**

A two-level design with 25–64-year-old women (*N* = 96′883), eligible for Pap smear screening, nested in 28 European countries, was used to analyze data from the European Health Interview Survey’s second wave, using multilevel logistic regression modelling.

**Results:**

Clear educational and income gradients in CCS uptake were found, which were smaller in countries with organized CCS programs, higher accessibility of the healthcare system and a higher level of decommodification. Furthermore, three-way interaction terms revealed that these gradients were smaller when organized CCS programs were implemented in countries with better accessibility of the healthcare system or a high level of decommodification.

**Conclusion:**

This study indicates that the combination of organized screening and high accessibility of the healthcare system or social protection is essential for having lower levels of inequality in CCS uptake. In such countries, the structural threshold for poorer and lower educated women to engage in CCS is lower. This may be explained by them having a better interaction with their GP, who may convince them of the screening test, lower out-of-pocket payments, and financial support to buffer against a disadvantageous position on the labor market.

**Supplementary Information:**

The online version contains supplementary material available at 10.1186/s12939-021-01548-6.

## Highlights

Organized programs do not always imply higher levels of screening uptake.

High accessibility of the healthcare system relates to lower inequalities in uptake.

High decommodification relates to lower inequalities in screening uptake.

These factors are essential for organized programs to relate to lower inequalities.

## Introduction

Since the introduction of cervical cancer screening (CCS) by means of Pap smears over 50 years ago, incidence and mortality rates have decreased, yet to a more substantial degree in countries where organized screening was implemented early, causing large variations across the European region [15]. Following these observations, the European Union has recommended 25–64-year-old women to undergo CCS on a 3-year interval to be organized in population-based programs [17]. In organized systems, screening-eligible women are systematically identified and personally invited to screen regularly, whereas, in systems with opportunistic screening, CCS is left to the initiative of physicians and women [8]. This institutional difference is referred to as the *screening strategy*. Organized systems are regarded to be more effective both in terms of participation and equity [2], as smaller education and income-based inequalities in CCS were observed in such systems compared to countries with opportunistic CCS [9, 23, 27]. These inequalities are referred to as the educational gradient and income gradient respectively.

However, some country-specific studies that investigated income and education-based inequalities in cancer screening participation before and after the implementation of an organized screening program generated mixed results on the inequality-reducing impact thereof [11, 13, 15, 28]. This variable impact over countries suggests that other institutional factors besides the screening strategy can be at stake, and may act as preconditions for organized programs to reduce inequalities [30]. We argue that two factors are crucial in this respect. Firstly, previous research on inequalities in female cancer screening from an institutional perspective has focused, besides the screening strategy, on the *accessibility of the healthcare system*, and reported that high accessibility increases the overall levels of CCS uptake [19] and diminishes inequalities therein, by decreasing barriers to women with lower income and lower education [9, 13, 31]. Secondly, previous research on the translation of social inequalities into health inequalities has focused on the welfare state level of *social protection* [3, 4, 14], yet, to the best of our knowledge, this macro-level institutional factor has not been studied in relation to inequalities in CCS uptake, but might be of strong importance as women’s financial barriers and social welfare dependency were shown to be related to CCS uptake [10, 21, 22]. These institutional characteristics have not been systematically assessed together in a European comparative context, but are essential for enhancing our understanding of CCS inequalities.

To sum up, the current research focuses on socioeconomic disparities in CCS uptake, whether these are moderated by the *screening strategy*, the broader *accessibility of the healthcare system*, the *social protection* redistribution mechanisms, and their dynamic interplay. The purpose of this study is to examine: (1) whether the magnitude of income and education-based inequalities in CCS uptake is different according to the accessibility of the healthcare system, and the broader level of social protection; (2) under which healthcare system access and social protection conditions an organized CCS program relates to lower levels of inequality in CCS uptake.

## Background

Several aspects relating to the accessibility of the healthcare system are particularly important concerning income and education-based inequalities in CCS uptake. The first concerns the availability of GPs. As CCS tests by means of Pap smears are performed either in a GP’s or a gynaecologist’s office or clinic, and GPs may act as gatekeepers for more specialized care [13], GPs have a central role in CCS uptake. Research indicated that high GP density per capita, is associated with a higher level of preventive care use [19], and that women who had consulted a GP within the last 12 months have a higher likelihood of having participated in CCS [8]. Moreover, GPs have been found not to discriminate based on socioeconomic position [26], implying lower inequalities in CCS uptake when GP consultations are widely available. Secondly, previous research has indicated that a high public investment in the health system and public share in health expenditures is associated with a higher level of preventive care use [19]. Moreover, the mix of public-private financing has proven an essential factor for income and education-based inequalities in screening uptake [9, 13, 19, 31], with larger inequalities in countries where healthcare financing mainly comes from private insurance and out-of-pocket payments [9, 13]. Based on this body of literature, contexts with a highly accessible healthcare system (high GP density, low out-of-pocket expenditure, and high public health expenditure) are expected to have lower income and education-based inequalities in CCS uptake compared to healthcare systems with lower accessibility.

Moreover, the accessibility of the healthcare system influences the success or failure of preventive programs [19]. In organized CCS systems, women in the target population receive an invitation letter to undergo a Pap smear test and are generally fully reimbursed for out-of-pocket expenses by the public health budget [2]. Nevertheless, often the appointment with the physician still needs to be made, and in some countries there are long waiting lists due to limited GP availability [20, 33], which is not favourable for lower educated women who may depend on meaningful GP interaction to be convinced of the screening test [1, 29]. Furthermore, engaging in preventive healthcare services may be perceived more costly for poorer women [26], especially in systems with high out-of-pocket payments (cost-sharing) and low public health expenditure [13, 19], even in countries with organized programs. Both factors taken together, in a context with low accessibility of the healthcare system, organized programs may not reach poorer and lower educated women. Following this reasoning, we hypothesize:

*Hypothesis 1: If the accessibility of the healthcare system is low, organized CCS is not associated with lower education- and income-based inequalities in CCS uptake*

Besides access to healthcare, systematic differences in health outcomes may be explained by the roles of the state in welfare provision and social protection at the macro level. The state’s general level of social protection through independence from the labor market has been captured in the concept of decommodification [16, 24]. Generous welfare states affect the relationship between socioeconomic position and health, by providing both social transfers (housing related benefits, unemployment replacement incomes for people who are inactive on the labor market, pensions, monetary, sickness and disability benefits) and key services (healthcare or social services) which are available for everyone, and hence can buffer deprivation risks [7, 14, 18]. Previous research has indicated that women of socioeconomically disadvantaged groups are less likely to screen for CC, many of whom were responsible for children at home, expressing financial barriers as reasons not to engage in screening, and who rely on social welfare [10, 21, 22]. Women with cumulative disadvantage especially depend on redistribution mechanisms and may be vulnerable under systems with low social protection. Based on this literature, higher decommodifying welfare states are expected to provide better protection against the disadvantageous health effects for women with a low socio-economic position, and to be related to lower levels of inequality in CCS uptake.

Furthermore, welfare states also impact the actual delivery of health services [3, 5], and it has been suggested that people are only able to turn incentives provided by the state into health benefits if they have the right economic resources at their disposal to do so [6, 7]. This is especially important for single mothers who may not receive substantial financial means in societies that depend on the male breadwinner model and not provide sufficient public welfare provision. Hence, even if CCS is readily available and offered through organized CCS programs, socioeconomically deprived women might continue to abstain from screening if the government does not take sufficient facilitating measures through social protection. Following this reasoning, we hypothesize that:

*Hypothesis 2: If the level of decommodification is low, organized CCS is not associated with lower education- and income-based inequalities in CCS uptake*

## Methods

### Sample

Data from the second wave of the repeated cross-sectional European Health Interview Survey (EHIS) were used, collected between 2013 and 2015. All EU member states, Norway, Iceland, and Switzerland are included in this study. Data for Switzerland were obtained from the Swiss Health Interview Survey (SHIS) 2012, gathered in 2011. Due to lacking information on the country-level degree of decommodification for Cyprus, Croatia and Lithuania, these countries were excluded from the analysis. In total, 28 countries were included. The macro-level data used to analyze differences in welfare state decommodification were obtained from the Organization for Economic Co-operation and Development (OECD) or aggregated from EU micro data, based on Israel and Spannagel [18]. For the construction of an index on the accessibility of the healthcare system, data were gathered from Eurostat (out-of-pocket expenditure as a percentage of total health expenditure), the World Health Organization (WHO) (public health expenditure as a percentage of GDP), and the United Nations Development Program (UNDP) (physician density per 10′000 people). For the other contextual characteristics, data were obtained from the UNDP (GDP per capita), the World Bank (Gini index) and the International Agency for Research on Cancer (IARC) (screening strategy). Sample age-ranges were defined according to the European screening recommendation guidelines [17]. That is, women aged 25–64. The final sample size consisted of 96′883 women (more detailed information on respondent selection in Additional file 1: Appendix A).

### Dependent variable

In the survey, respondents were asked when they had last had a cervical smear test. Possible answers were: within the past 12 months, 1 to less than 2 years ago, 2 to less than 3 years ago, more than 3 years ago, or never. Following the recommended three-year screening interval, proposed in the European recommendation guidelines, these answer categories were recoded measuring whether the respondent was screened within the past 3 years (yes = 1, no = 0).

### Independent variables

As predictors of interest, educational attainment and monthly household income were included at the individual level as indicators of socioeconomic position. At the macro level we included the country’s screening strategy, accessibility of the healthcare system, and level of decommodification. *Educational attainment* was measured as the highest level of education completed, based on the ISCED-2011 classification and was recoded into a variable with three categories: low education (0) based on ISCED 0–2; middle education (1) based on ISCED 3–4; high education (2) based on ISCED 5–8. For *household income*, the net monthly equivalized income of the household was used, and was coded: below 1st quintile (0), between 1st and 2nd quintile (1), between 2nd and 3rd quintile (2), between 3rd and 4th quintile (3), between 4th and 5th quintile (4). At the macro-level, a country’s *screening strategy* was classified in one of these three categories in accordance to [30]: opportunistic (0) if no formal program was available at the time the survey was conducted, regional/rollout ongoing (1) if the implementation of a program had started or was available only in some regions, organized (2) if a population-based program was readily available for the entire country. Further, to analyze the magnitude of educational and income-based disparities in screening uptake according to the accessibility of the healthcare system, an *access to healthcare index* was constructed by taking the sum of countries’ scores on factors that had proven relevant for preventive healthcare services uptake [19]: out-of-pocket expenditure as a percentage of total health expenditure, public health expenditure as a percentage of GDP, and GP density per 10′000 inhabitants (Additional file 1: Appendix B Table 1). These three parameters were given the same weight, and out-of-pocket expenditure was reversed to match the direction of the other two aspects in this index. A low score on this index corresponds with low accessibility of the healthcare system, and a high score with high accessibility. Furthermore, to analyze the magnitude of educational and income-based disparities in screening uptake in consonance with the broader welfare state degree of social protection, the *decommodification index* was used [16, 24]. This composite index is based on the unemployment benefits index, the social assistance index and the health provision index [18], providing an image of the degree to which people in a certain country are socially protected by the state or dependent from the labor market (data retrieved from Israel and Spannagel [18]). The decommodification index was divided by 10 to make the results more readable, with a low score corresponding with a low level of decommodification, and vice versa.

To control for acknowledged associations with CCS, the following covariates were taken into account, at the individual level: age, marital status, urbanity, self-rated health, work status, country of birth, and time since last GP visit [8, 12, 27]. At the macro level we controlled for income inequality by including the *Gini coefficient* ranging from 0 (total income equality) to 100 (total income inequality), and the general level of economic development by including *GDP* per capita (divided by 100 to make the results more readable). All continuous macro-level variables were grand mean centered.

### Statistical analyses

**Fig. 1:**
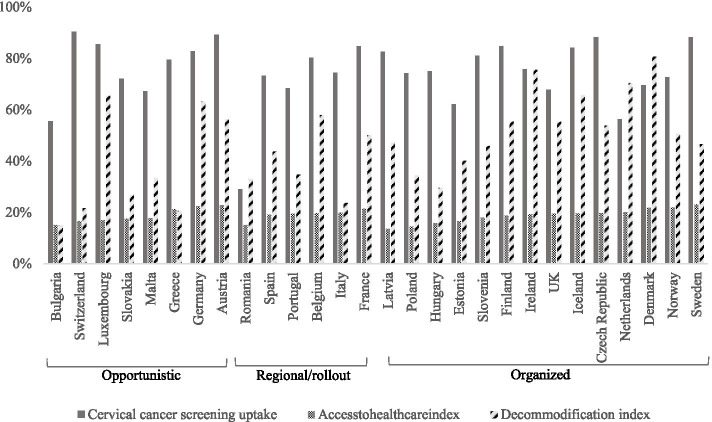
Cervical cancer screening uptake in women aged 25–64 by country, screening strategy, accessibility of the healthcare system and degree of decommodification

**Fig. 2.**
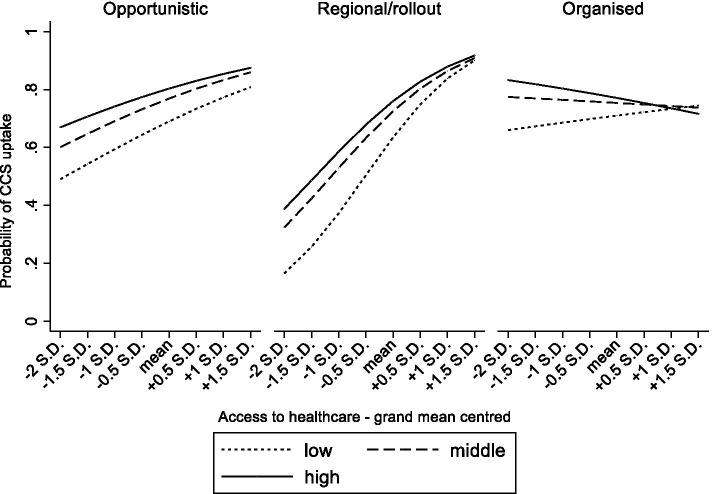
From Table 3 model 3 Predicted probabilities of cervical cancer screening uptake in women aged 25–64 by educational attainment, screening strategy and accessibility of the healthcare system.

**Fig. 3:**
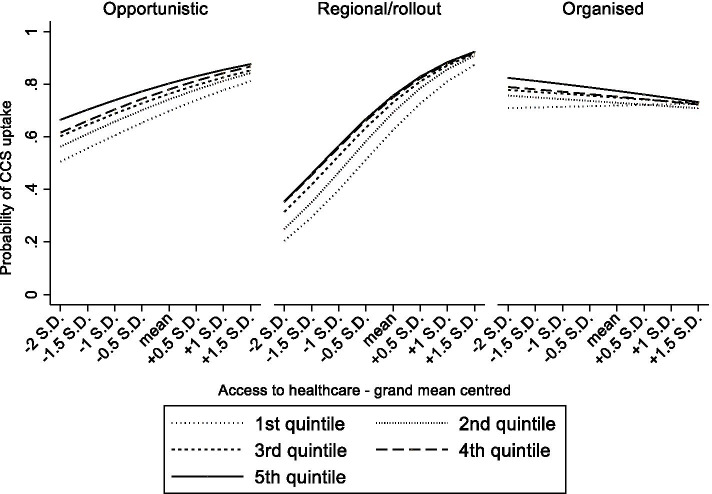
From Table 3 model 4 Predicted probabilities of cervical cancer screening uptake in women aged 25–64 by household income, screening strategy and accessibility of the healthcare system.

**Fig. 4:**
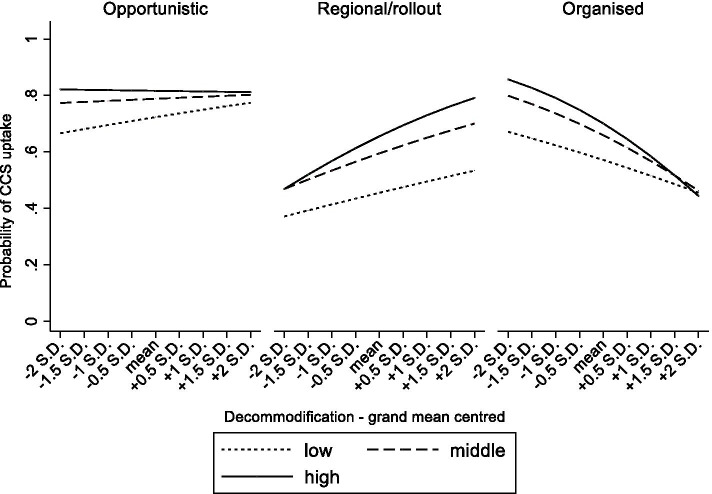
From Table 3 model 7 Predicted probabilities of cervical cancer screening uptake in women aged 25–64 by educational attainment, screening strategy and degree of decommodification.

**Fig. 5:**
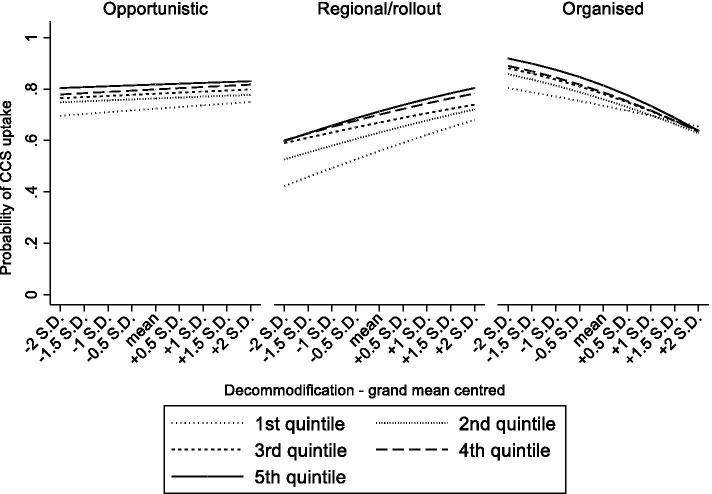
From Table 3 model 8 Predicted probabilities of cervical cancer screening uptake in women aged 25–64 by household income, screening strategy and degree of decommodification.

Firstly, descriptive statistics and proportions of CCS uptake were calculated for the individual-level predictors of interest and country-level variables, shown in Table 1 and Fig. 1. Secondly, we performed multi-level logistic regressions, which allows for possible similarities between women living in the same country [25]. The multi-level models consisted of two levels: 96′883 women nested in 28 countries and were built stepwise. In Table 2, the first model estimated the main effects of educational attainment and household income to get an image of the educational and income inequalities in CCS uptake. In the second model, the macro-level variables were added. The third and fourth models examined the magnitude of educational and income-based inequalities in CCS uptake according to the screening strategy, by adding two-way cross-level interaction terms. Next, in Table 3, the size of socioeconomic disparities was brought in relation to the accessibility of the healthcare system (models 1–4) and the level of decommodification (models 5–8). We first estimated the two-way interactions with the socioeconomic indicators for both factors and then assessed the relative impact of the screening strategy on the magnitude of inequalities in screening uptake by adding three-way interaction terms. In all models, coefficients were adjusted for the individual and country-level control variables. Odds ratios (OR) and degrees of significance are shown. Lastly, the two-way (Additional file 1: Appendix C Figures 1-4)) and three-way interactions (Figs. 2, 3, 4 and 5) were plotted graphically to facilitate the interpretation of the interaction terms. Analyses were conducted with SPSS 22 and STATA 15. The variance inflation factors (VIF) were below 3 for all variables, suggesting no problems of multicollinearity.

**Table 1:** Descriptive statistics for Pap smear uptake, among 96′883 women aged 25–64 in the European Health Interview Survey

	N(%)	No uptake %	Uptake %	Sig.^c^
***Individual level***				
Education				***
low	21′904 (22.6)	37.7	62.3	
middle	43′274 (44.7)	24.3	75.7	
High	31′705 (32.7)	18.4	81.6	
Income				***
1st quintile	17′374 (17.9)	34.4	65.6	
2nd quintile	17′530 (18.1)	28.0	72.0	
3rd quintile	19′092 (19.7)	24.4	75.6	
4th quintile	21′277 (22.0)	22.0	78.0	
5th quintile	21′610 (22.3)	20.3	79.7	
***Country level***				
Screening strategy				***
Opportunistic	29′695 (30.6)	17.5	82.5	
Regional/rollout	31′059 (32.1)	33.0	67.0	
Organized	36′129 (37.3)	25.4	74.6	
	Mean	S.D.	Min	Max
ATH^a^	0.135	2.668	−5.340	4.165
Decommodification^b^	0.020	1.551	−3.020	3.554

^a^Access to healthcare; ^b^Welfare state decommodification; ^c^Pearson Chi^2^

*p < 0.05; **p < 0.01; ***p < 0.001

**Table 2:** Multilevel logistic regression coefficients and cross-level interaction effects in cervical cancer screening uptake by screening strategy, in women aged 25–64 (*N* = 96′883) in the European Health Interview Survey

	Model 1		Model 2		Model 3		Model 4	
	OR	Sig.	OR	Sig.	OR	Sig.	OR	Sig.
***Individual level***								
Education (low)								
Middle	1.47	***	1.47	***	1.54	***	1.45	***
High	1.71	***	1.71	***	1.87	***	1.69	***
Income (1st quintile)								
2nd quintile	1.25	***	1.25	***	1.25	***	1.27	***
3rd quintile	1.45	***	1.45	***	1.45	***	1.43	***
4th quintile	1.57	***	1.57	***	1.56	***	1.6	***
5th quintile	1.74	***	1.74	***	1.72	***	1.84	***
***Country level***								
Screening strategy (opportunistic)								
Regional/rollout			0.75		0.75		0.67	
Organized			0.90		1.05		1.04	
ATH^a^			1.09		1.09		1.09	
Decommodification^b^			0.91		0.91		0.91	
***Cross-level interactions***								
Education (low)*screening strategy								
middle*regional/rollout					1.01			
middle*organized					0.85	**		
high*regional/rollout					1.00			
high*organized					0.79	***		
Income (1st quintile)*screening strategy								
2nd quintile*regional/rollout							1.09	
2nd quintile*organized							0.88	*
3rd quintile*regional/rollout							1.20	**
3rd quintile*organized							0.88	*
4th quintile*regional/rollout							1.20	**
4th quintile*organized							0.80	***
5th quintile*regional/rollout							1.08	
5th quintile*organized							0.81	***

*Note*: All models are adjusted for age, marital status, urbanity, self-rated health, work status, country of birth, time since last GP visit, GDP per capita and Gini

*p < 0.05; **p < 0.01; ***p < 0.001

^a^Access to healthcare; ^b^Welfare state decommodification

**Table 3:** Multilevel logistic regression coefficients and cross-level interaction effects in cervical cancer screening uptake by macro-level access to healthcare and decommodification, in women aged 25–64 (*N* = 96′883) in the European Health Interview Survey

	Access to healthcare								Decommodification							
	Model 1		Model 2		Model 3		Model 4		Model 5		Model 6		Model 7		Model 8	
	OR	Sig.	OR	Sig.	OR	Sig.	OR	Sig.	OR	Sig.	OR	Sig.	OR	Sig.	OR	Sig.
***Individual level***																
Education (low)																
Middle	1.49	***	1.47	***	1.56	***	1.45	***	1.46	***	1.45	***	1.50	***	1.45	***
High	1.74	***	1.71	***	1.96	***	1.69	***	1.72	***	1.69	***	1.84	***	1.68	***
Income (1st quintile)																
2nd quintile	1.25	***	1.25	***	1.24	***	1.28	***	1.25	***	1.24	***	1.25	***	1.27	**
3rd quintile	1.44	***	1.45	***	1.43	***	1.45	***	1.44	***	1.44	***	1.43	***	1.43	**
4th quintile	1.56	***	1.57	***	1.54	***	1.61	***	1.55	***	1.55	***	1.54	***	1.61	***
5th quintile	1.71	***	1.73	***	1.69	***	1.88	***	1.71	***	1.72	***	1.70	***	1.84	***
***Country level***																
Screening strategy (opportunistic)																
Regional/rollout	0.75		0.75		0.76		0.69		0.74		0.74		0.66		0.67	
Organized	0.90		0.91		1.11		1.12		0.90		0.91		1.03		1.09	
ATH^a^	1.14	**	1.10		1.19	**	1.19	**	1.09		1.09		1.12	*	1.12	*
Decommodification^b^	0.91		0.91		0.88		0.88		0.97		0.98		1.10		1.05	
***Cross-level interactions***																
Education (low)*screening strategy																
Middle*regional/rollout					1.02								1.09			
Middle*organized					0.82	***							0.96			
High*regional/rollout					1.00								1.14			
High*organized					0.73	***							0.95			
Income (1st quintile)*screening strategy																
2nd quintile*regional/rollout							1.10								1.07	
2nd quintile*organized							0.83	**							0.92	
3rd quintile*regional/rollout							1.19	*							1.14	
3rd quintile*organized							0.82	**							0.94	
4th quintile*regional/rollout							1.20	**							1.18	*
4th quintile*organized							0.75	***							0.87	*
5th quintile*regional/rollout							1.07								1.09	
5th quintile*organized							0.74	***							0.93	
Education (low)*ATH|decommodification																
middle*ATH|decommodification	0.96	***			0.99				0.94	***			0.93	**		
high*ATH|decommodification	0.93	***			0.97				0.90	***			0.89	***		
Income (1st quintile)*ATH|decommodification																
2nd quintile*ATH|decommodification			1.00				1.00				0.94	***			0.98	
3rd quintile*ATH|decommodification			0.99				0.99				0.92	***			0.99	
4th quintile*ATH|decommodification			0.99				0.99				0.91	***			0.99	
5th quintile*ATH|decommodification			0.97	**			0.98				0.90	***			0.98	
Screening strategy (opportunistic)*ATH|decommodification																
Regional/rollout*ATH|decommodification					1.32	*	1.25						0.99		1.13	
Organized*ATH|decommodification					0.88		0.85						0.79		0.83	
Education (low)*screening strategy (opportunistic) *ATH|decommodification																
Middle*regional/rollout*ATH|decommodification					0.92	**							1.14	**		
Middle*organized*ATH|decommodification					0.94	**							0.96			
High*regional/rollout*ATH|decommodification					0.92	**							1.29	***		
High*organized*ATH|decommodification					0.91	***							0.93	*		
Income (1st quintile)*screening strategy (opportunistic)*ATH|decommodification																
20032nd quintile*regional/rollout*ATH|decommodification							1.01								1.07	
2nd quintile*organized*ATH|decommodification							0.96								0.93	
3rd quintile*regional/rollout*ATH|decommodification							1.00								1.14	
3rd quintile*organized*ATH|decommodification							0.97								0.94	
4th quintile*regional/rollout*ATH|decommodification							0.97								1.18	*
4th quintile*organized*ATH|decommodification							0.95	*							0.87	*
5th quintile*regional/rollout*ATH|decommodification							0.99								1.09	
5th quintile*organized*ATH|decommodification							0.95	*							0.93	

*Note*: All models are adjusted for age, marital status, urbanity, self-rated health, work status, country of birth, time since last GP visit, GDP per capita and Gini

*Note*: The “ATH|decommodification” label is used to differentiate between models on access to healthcare and models on welfare state decommodification

*p < 0.05; **p < 0.01; ***p < 0.001

^a^Access to healthcare; ^b^Welfare state decommodification

### Sensitivity analyses

Three sensitivity analyses were performed. Detailed information is available in Additional file 1: Appendix D.

## Results

### Pap smear uptake according to women’s characteristics and macro-level determinants

Table 1 shows higher CCS coverage among women with high education (81.6%) compared to women with middle (75.7%) or low (62.3%) levels of education. Furthermore, screening uptake was consistently higher according to higher household income, ranging from 65.6% in the poorest women to 79.7% in the richest. Figure 1 shows the participation rates for CCS by screening strategy, access to healthcare and level of decommodification. There is large variation over countries in terms of CCS participation, with the highest rates in Switzerland (90.4%) and Austria (89.2%), and lowest in Romania (29.0%) and Bulgaria (55.4%). Half of the countries have an organized screening strategy. On average, CCS appeared to be highest in countries with an opportunistic screening strategy (82.5%), followed by countries with organized screening programs (74.6%) and regional/rollout strategies (67.0%) (Table 1). The accessibility of the healthcare system was found to be lowest in Latvia (− 5.3) and Poland (− 4.4), and highest in Sweden (4.2) and Austria (3.9). Further, the degree of decommodification was lowest in Bulgaria (− 3.0) and Greece (− 2.4), and highest in Denmark (3.6) and Ireland (3.0).

### Educational and income inequalities in screening uptake

Tables 2 and 3 show the results from the multilevel logistic regression models. As can be seen in Table 2 model 1, the likelihood of having had a CCS test within the past 3 years was significantly higher as women had a higher level of education (Table 2, OR_model1_ middle = 1.47, high = 1.71), compared to the lower educated. A similar phenomenon was found for household income, with significantly more screening uptake among women with a higher household income (Table 2, OR_model1_ 2nd quintile = 1.25, 3rd quintile = 1.45, 4th quintile = 1.57, 5th quintile = 1.74) compared to women with a lower household income. So, clear gradients were observed for both education and income.

### Magnitude of inequalities by context level screening strategy, access to healthcare and decommodification

However, the magnitude of these educational and income gradients was moderated by the screening strategy and countries’ levels of healthcare access and degree of decommodification. Firstly, inequalities in CCS uptake based on education (Table 2, OR_model3_ middle*organized = 0.85, high*organized = 0.79) and income (Table 2, OR_model4_ 2nd quintile*organized = 0.88, 3rd quintile*organized = 0.88, 4th quintile*organized = 0.80, 5th quintile*organized = 0.81) were smaller in organized systems compared to opportunistic systems. Remarkably, when only regional programs were available or the program was being rolled out, larger income inequalities were observed (Table 2, OR_model4_ 3rd quintile*regional/rollout = 1.20, 4th quintile*regional/rollout = 1.20). Secondly, a highly accessible healthcare system corresponded with smaller education-based differences in screening participation (Table 3, OR_model1_ middle*ATH = 0.96, high*ATH = 0.93; Additional file 1: Appendix Figure 1). For household income, a significant moderation of the gap was observed only between the lowest and highest income quintiles (Table 3, OR_model2_ 5th quintile*ATH = 0.97; Additional file 1: Appendix Figure 2). Thirdly, a high level of decommodification was associated with lower inequalities in CCS uptake based on education (Table 3, OR_model5_ middle*decommodification = 0.94, high*decommodification = 0.90; Additional file 1: Appendix Figure 3) and income (Table 3, OR_model6_ 2nd quintile*decommodification = 0.94, 3rd quintile*decommodification = 0.92, 4th quintile*decommodification = 0.91, 5th quintile*decommodification = 0.90; Additional file 1: Appendix Figure 4).

### Organized CCS, and the context-dependency of its impact

Next, to test the interplay between the screening strategy and broader contextual determinants, several three-way cross-level interaction terms were estimated. Firstly, in a context with a highly accessible healthcare system, organized CCS related to lower disparities in CCS between the lower and higher educated (Table 3, OR_model3_, middle*organized*ATH = 0.94, high*organized*ATH = 0.91). A similar phenomenon was found for systems with only regional programs or where the rollout was ongoing (Table 3, OR_model3_, middle*regional/rollout*ATH = 0.92, high*regional/rollout*ATH = 0.92). For household income, a highly accessible healthcare system was associated with lower inequalities if screening was organized (Table 3, OR_model4_, 4th quintile*organized*ATH = 0.95, 5th quintile*organized*ATH = 0.95). These interaction terms are visually presented in Figs. 2 and 3. They show that highly accessible healthcare systems come with smaller educational and income disparities in screening uptake, and that this is even stronger when countries have also implemented organized CCS screening. Secondly, in a context of high decommodification, organized CCS corresponded with lower educational inequalities in CCS uptake (Table 3, OR_model7_, high*organized*decommodification = 0.93). Remarkably, educational inequalities were larger in systems with high decommodification and regional programs or where the rollout was ongoing (Table 3, OR_model7_, middle*regional/rollout*decommodification = 1.14, high*regional/rollout*decommodification = 1.29). Turning to household income, in a context of high decommodification, the gap in CCS uptake between women with lower versus higher incomes appeared to be significantly smaller when countries had organized CCS compared to opportunistic CCS (Table 3, OR_model8_ 4th quintile*organized*decommodification = 0.87). The reverse was found in systems with only regional programs or where the rollout was ongoing (Table 3, OR_model8_ 4th quintile*regional/rollout*decommodification = 1.18). These interaction terms are graphically displayed in Figs. 4 and 5. As further illustrated in these figures, organized CCS only resulted in lower levels of inequality in screening uptake when decommodification was high.

## Discussion

The current research contributes to previous CCS literature by assessing the context dependency of the previously found basic-relation between organized screening and inequalities in screening uptake. This study aimed at assessing whether the magnitude of education- and income-based inequalities in CCS uptake is different according to the macro-level accessibility of the healthcare system and level of social protection (decommodification), and under which of these macro-level conditions organized CCS can relate to lower levels of inequality therein.

Several interesting phenomena were uncovered. Firstly, in line with previous literature [8, 23, 27], clear educational and income gradients were found in CCS uptake, with women with higher educational attainment and higher household income showing consistently higher levels of screening uptake compared to their counterparts. Secondly, it appeared that organized CCS does not necessarily correspond with higher levels of CCS uptake. This may be explained by CCS overuse, which might be less present in contexts with organized screening programs than in opportunistic screening contexts [12]. Thirdly, we found that education- and income-based disparities in CCS uptake were smaller in countries with organized CCS, a higher accessibility of the healthcare system and higher levels of decommodification. This is in line with our expectations based on previous research [9, 13, 31]. Fourthly, to go beyond inconsistent evidence across European countries [11, 13, 28], we examined the context-dependency of the impact of the screening strategy on inequalities in screening uptake. Whereas educational inequalities in CCS participation were smaller in countries with a high score on the access to healthcare-index, the moderation effect of access to healthcare was stronger if these countries had also implemented organized CCS. Hence, the combination of a high accessibility to healthcare and the presence of an organized screening program was associated with smaller differences in participation between women with low and high levels of education. This was less pronounced for income-inequality (no gradual reduction), but the results nevertheless show that the gap between the lowest income quintile and the highest two quintiles was significantly smaller in countries that had a high accessibility to healthcare, and even smaller if these countries also had an organized screening program. These findings provide supportive evidence for hypothesis 1. Further, a similar phenomenon was observed concerning the level of decommodification. Whereas educational and income inequalities in CCS uptake were smaller in countries with a high level of decommodification, this moderation was stronger if these countries had also implemented organized CCS. Hence, the combination of both factors leads to even smaller levels of inequality. On the other hand, if countries have low levels of decommodification, inequalities are larger, irrespective of the type of CCS program. Based on these findings we can confirm hypothesis 2. Lastly, in countries where only a regional program was available or where the rollout was ongoing, the accessibility of the healthcare system and level of decommodification appeared to have a reverse impact compared to systems with organized CCS, that is, larger socioeconomic inequalities in CCS uptake. Assessing this in-between situation was beyond the scope of the current research. Nevertheless, a possible explanation for this phenomenon may be that women with higher health literacy would get involved in new (screening) technologies in a more timely manner and would consequently adapt this innovation when it is still in an earlier stage of diffusion [32].

Some key limitations of this study must be noted. Firstly, in the EHIS, no information for the reasons why women engaged in CCS is available, so no distinction could be made between screening for preventive or diagnostic reasons. Crucial information on incentives for Pap smear uptake is missing, and we could not determine at the individual level whether or not a woman screened within the framework of an organized program. Secondly, choices had to be made concerning the context-level indicators. Besides the indexes on accessibility of the healthcare system and decommodification, some other aspects that would support preventive healthcare behaviour in a certain household via monetary incentives, such as healthcare policy mechanisms that assist households through cost-reimbursement of preventive healthcare services, might impact levels of CCS uptake [12]. Thirdly, by combining macro- and individual-level data, we cannot exclude that findings may suffer from ecological fallacy. By accounting for population heterogeneity and confounding factors, this bias may remain, however, at an acceptable level. Moreover, we assumed that macro social factors are ubiquitous in their influence on the general population, which is a theoretical assumption. It is probable that macro-level factors do not have the same influence across subgroups. Lastly, as a cross-country European-wide research design was used, we may not deduce conclusions to individual countries, and can only interpret the findings in general terms.

## Conclusion

In the current study, we found that organized screening programs do not always imply higher levels of screening uptake. In line with previous literature [3, 19, 31], we highlighted high accessibility of the healthcare system and social protection by the welfare state to be crucial institutional factors in terms of inequalities in CCS uptake. We brought these institutional factors in relation to European countries’ cancer screening strategies. It appears that in states with high accessibility to healthcare (through high public health expenditure, low out-of-pocket payments, and high GP density) and high social protection (though low financial dependence from the labor market) the structural threshold for women with a lower socioeconomic position to engage in CCS is lower, resulting in lower levels of inequality in CCS uptake. Based on our findings, some policy implications for reducing barriers to socioeconomically deprived women can be suggested. Firstly, as the most vulnerable women such as those with the lowest levels of income and education may depend on meaningful interaction with available GPs to convince them of engaging in CCS [1, 29], facilitating GP contact should be incorporated in organized programs. The transportation cost to go on consultation should be limited in order to make this easier for women living in areas with few available GPs, and to support the financially most vulnerable. Also, physicians should be made more aware of the social discrepancy in CCS uptake, in order to further reduce discriminatory practices and the psychosocial barriers socioeconomically deprived women encounter [21]. Secondly, the actual appointment for the screening test should already be made when sending women the invitation letter, to bypass reluctance. For single mothers, childcare services may be provided on the day of the appointment, and they could be granted a day of sick-leave to avoid loss of income. Lastly, screening tests should be offered completely out of charge, further reducing the share of the out-of-pocket payment, once every 3 years in relation to the European guidelines.

We can conclude that organized programs should not only focus on the general level of screening uptake, but should also take measures to reduce the level of inequality therein, relating to both access to healthcare and social protection.

## Supplementary Information

**Additional file 1.**

## Data Availability

The data that support the findings of this study are available from Eurostat but restrictions apply to the availability of these data, which were used under license for the current study, and so are not publicly available.

## References

[1] Arbyn M, Fabri V, Temmerman M, Simoens C (2014). Attendance at cervical cancer screening and use of diagnostic and therapeutic procedures on the uterine cervix assessed from individual health insurance data (Belgium, 2002-2006). PLoS One.

[2] Arbyn M, Rebolj M, De Kok IM, Fender M, Becker N, O’Reilly M, Andrae, B. J. E. j. o. c. (2009). The challenges of organising cervical screening programmes in the 15 old member states of the. European Union.

[3] Bambra C (2005). Cash versus services:‘worlds of welfare’and the decommodification of cash benefits and health care services. J Soc Policy.

[4] Bambra C (2007). Going beyond the three worlds of welfare capitalism: regime theory and public health research. J Epidemiol Community Health.

[5] Bambra C, Fox D, Scott-Samuel A (2005). Towards a politics of health. Health Promot Int.

[6] Bartley M (2003). Health inequality and societal institutions. Soc Theory Health.

[7] Beckfield J, Bambra C, Eikemo TA, Huijts T, McNamara C, Wendt C (2015). An institutional theory of welfare state effects on the distribution of population health. Soc Theory Health.

[8] Burton-Jeangros C, Cullati S, Manor O, Courvoisier DS, Bouchardy C, Guessous I (2017). Cervical cancer screening in Switzerland: cross-sectional trends (1992–2012) in social inequalities. Eur J Pub Health.

[9] Carrieri V, Wübker A (2013). Assessing inequalities in preventive care use in Europe. Health policy.

[10] Catarino RR, Vassilakos PP, Royannez-Drevard II, Guillot CC, Alzuphar SS, Fehlmann AA, Petignat PP (2016). Barriers to cervical cancer screening in Geneva (DEPIST study). J Lower Genital Tract Dis.

[11] Cullati S, von Arx M, Courvoisier DS, Sandoval JL, Manor O, Burton-Jeangros C, Swiss N. Organised population-based programmes and change in socioeconomic inequalities in mammography screening: A 1992-2012 nationwide quasi-experimental study. Prev Med. 2018;116:19-26. 10.1016/j.ypmed.2018.08.012. 10.1016/j.ypmed.2018.08.012 30145347

[12] De Prez V, Jolidon V, Willems B, Cullati S, Burton-Jeangros C, Bracke P (2020). Cervical cancer (over) screening in Belgium and Switzerland: trends and social inequalities. Eur J Pub Health.

[13] Devaux M (2015). Income-related inequalities and inequities in health care services utilisation in 18 selected OECD countries. Eur J Health Econ.

[14] Eikemo TA, Bambra C, Joyce K, Dahl E (2008). Welfare state regimes and income-related health inequalities: a comparison of 23 European countries. Eur J Public Health.

[15] Elfström KM, Arnheim-Dahlström L, von Karsa L, Dillner J (2015). Cervical cancer screening in Europe: quality assurance and organisation of programmes. Eur J Cancer.

[16] Esping-Andersen G (1990). The three worlds of welfare capitalism: Princeton University press.

[17] European Commission. Council recommendation of 2 December 2003 on Cancer screening (2003/878/EC) 878. Off J Eur Union. 2003:34–8.

[18] Israel S, Spannagel D (2019). Material deprivation in the EU: a multi-level analysis on the influence of decommodification and defamilisation policies. Acta Sociol.

[19] Jusot F, Or Z, Sirven N (2012). Variations in preventive care utilisation in Europe. Eur J Ageing.

[20] Kivistik A, Lang K, Baili P, Anttila A, Veerus P (2011). Women's knowledge about cervical cancer risk factors, screening, and reasons for non-participation in cervical cancer screening programme in Estonia. BMC Womens Health.

[21] McLachlan E, Anderson S, Hawkes D, Saville M, Arabena K (2018). Completing the cervical screening pathway: factors that facilitate the increase of self-collection uptake among under-screened and never-screened women, an Australian pilot study. Curr Oncol.

[22] Menvielle G, Richard J-B, Ringa V, Dray-Spira R, Beck F (2014). To what extent is women’s economic situation associated with cancer screening uptake when nationwide screening exists? A study of breast and cervical cancer screening in France in 2010. Cancer Causes Control.

[23] Palència L, Espelt A, Rodríguez-Sanz M, Puigpinós R, Pons-Vigués M, Pasarín MI, Borrell C (2010). Socio-economic inequalities in breast and cervical cancer screening practices in Europe: influence of the type of screening program. Int J Epidemiol.

[24] Scruggs L, Allan J (2006). Welfare-state decommodification in 18 OECD countries: a replication and revision. J Eur Soc Policy.

[25] Snijders TA, Bosker RJ (2011). Multilevel analysis: an introduction to basic and advanced multilevel modeling: sage.

[26] Van Doorslaer E, Masseria C, Koolman X (2006). Inequalities in access to medical care by income in developed countries. Cmaj.

[27] Willems B, Bracke P (2018). The education gradient in cancer screening participation: a consistent phenomenon across Europe?. Int J Public Health.

[28] Willems B, Bracke P (2018). The impact of regional screening policies on the diffusion of cancer screening participation in Belgium: time trends in educational inequalities in Flanders and Wallonia. BMC Health Serv Res.

[29] Willems B, Bracke P (2018). Participants, physicians or Programmes: participants’ educational level and initiative in cancer screening. Health Policy.

[30] Willems B, Cullati S, Prez VD, Jolidon V, Burton-Jeangros C, Bracke P (2020). Cancer screening participation and gender stratification in Europe. J Health Soc Behav.

[31] Wübker A (2014). Explaining variations in breast cancer screening across European countries. Eur J Health Econ.

[32] Zapata-Moya ÁR, Willems B, Bracke P (2019). The (re) production of health inequalities through the process of disseminating preventive innovations: the dynamic influence of socioeconomic status. Health Sociol Rev.

[33] Zodzika J, Krumina K, Jermakova I, Kojalo U, Plisko O, Santare D, Lazdane G (2021). Post-reproductive aged women: a lost generation in the cervical cancer screening programme. Eur J Contracept Reprod Health Care.

