# Supplementary material for: Cervical cancer screening programs and their context-dependent effect on inequalities in screening uptake: a dynamic interplay between public health policy and welfare state redistribution
Source: Int J Equity Health. 2021 Sep 24;20:211. doi: 10.1186/s12939-021-01548-6 (PMC8464130; doi:10.1186/s12939-021-01548-6)
Supplement: Supplementary file 1 — Additional file 1. [file 12939_2021_1548_MOESM1_ESM.docx]

**APPENDIX A:** **respondent selection**

The initial sample consisted of 322'321 respondents. After omitting men (N=148'219; 46%), women outside the screening-eligible age (N=17'610; 10.1% below 25 and N=47'113; 30.1% above 64), and students (N=1'796; 1.6%) to make sure that all women in our sample had completed their education in order to cancel-out ambiguity on a variable on educational attainment, the sample consisted of N=107'583 women. After further omitting cases with missing information on CCS uptake (N=4'502; 4.2%), and the key predictors on educational attainment (N=486; 0.5%) and household income (N=5'712; 5.6%), the final sample size consisted of 96'883 women aged 25-64.

**APPENDIX B: Construction of access to healthcare index**

| **Appendix Table 1** Parameters used to construct access to healthcare index | | | |
| --- | --- | --- | --- |
| **Country** | **Out-of-pocket expenditure as % of total health expenditure (2013)** | **Public health expenditure as % of GDP (2013)** | **Physician density per 10’000 inhabitants (2007-2017)** |
| Austria | 19.2 | 7.4 | 52.3 |
| Belgium | 18.0 | 8.0 | 30.1 |
| Bulgaria | 47.0 | 4.0 | 40.0 |
| Czech Republic | 13.6 | 6.5 | 36.8 |
| Denmark | 13.8 | 8.6 | 36.6 |
| Estonia | 22.6 | 4.5 | 34.3 |
| Finland | 18.9 | 6.8 | 32.0 |
| France | 9.9 | 8.7 | 32.4 |
| Germany | 13.1 | 8.3 | 41.9 |
| Greece | 33.7 | 5.2 | 62.6 |
| Hungary | 28.4 | 4.8 | 30.9 |
| Iceland | 18.3 | 6.6 | 37.9 |
| Ireland | 14.3 | 7.3 | 29.6 |
| Italy | 21.8 | 6.8 | 40.2 |
| Latvia | 38.5 | 3.2 | 32.1 |
| Luxembourg | 11.0 | 4.8 | 29.2 |
| Malta | 36.8 | 6.0 | 39.1 |
| Netherlands | 11.5 | 7.1 | 34.8 |
| Norway | 14.6 | 7.6 | 43.9 |
| Poland | 23.6 | 4.5 | 22.9 |
| Portugal | 27.0 | 6.1 | 44.3 |
| Romania | 20.2 | 4.1 | 26.7 |
| Slovakia | 23.3 | 5.5 | 34.5 |
| Slovenia | 13.0 | 6.2 | 28.2 |
| Spain | 24.0 | 6.4 | 38.7 |
| Sweden | 15.5 | 9.2 | 41.9 |
| Switzerland | 28.3 | 3.6 | 42.5 |
| UK | 15.0 | 7.8 | 28.3 |

Note: Source of data

Out-of-pocket expenditure as % of total health expenditure: Eurostat

Public health expenditure as % of GDP: World Health Organization (WHO)

Physician density per 10’000 inhabitants: United Nations Development Program (UNDP)

**APPENDIX C: graphical representation of two-way interactions**

**Figure 1, from Table 3 model 1** Predicted probabilities of cervical cancer screening uptake in women aged 25-64 by educational attainment and accessibility of the healthcare system

**Figure 2, from Table 3 model 2** Predicted probabilities of cervical cancer screening uptake in women aged 25-64 by household income and accessibility of the healthcare system

**Figure 3, from Table 3 model 5** Predicted probabilities of cervical cancer screening uptake in women aged 25-64 by educational attainment and degree of decommodification

**Figure 4, from Table 3 model 6** Predicted probabilities of cervical cancer screening uptake in women aged 25-64 by household income and degree of decommodification

**APPENDIX D: sensitivity analyses**

Three sensitivity analyses were performed. Firstly, as Romania appeared to be an influential case, analyses were also conducted without this country (Appendix Table 2). Generally, the results remained the same, although two noteworthy differences were observed. Without Romania, in case of high accessibility of the healthcare system and a regional/rollout CCS strategy, educational inequalities in CCS uptake were no longer lower (this was also observed in the two-way interaction) and income-based inequalities therein were higher, compared to contexts with opportunistic CCS. Further, in case of high decommodification, income-based inequalities in CCS uptake were even smaller under organized CCS, and showed a clear diminishing gradient. Secondly, models were estimated with the cross-level interactions for access to healthcare and decommodification together (Appendix Table 3). From the two-way interactions it appeared that, in case of low accessibility of the healthcare system, a high level of decommodification has strong income-based inequality-reducing effects. This also appears from the three-way interactions, where in a context with low accessibility of the healthcare system, income inequalities in CCS are much lower in case of a combination of organized CCS and a high level of decommodification. Thirdly, the two-way cross-level interaction terms were re-estimated using fixed-effects models, and generated similar results (not shown).

| **Appendix Table 2** Multilevel logistic regression coefficients and cross-level interaction effects in cervical cancer screening uptake by macro-level access to healthcare and decommodification, in women aged 25-64 (N=91’566) in the European Health Interview Survey, without Romania | | | | | | | | | | | | | | | | |
| --- | --- | --- | --- | --- | --- | --- | --- | --- | --- | --- | --- | --- | --- | --- | --- | --- |
|  | Access to healthcare | | | | | | | | Decommodification | | | | | | | |
|  | Model 1 | | Model 2 | | Model 3 | | Model 4 | | Model 5 | | Model 6 | | Model 7 | | Model 8 | |
|  | OR | Sign | OR | Sign | OR | Sign | OR | Sign | OR | Sign | OR | Sign | OR | Sign | OR | Sign |
| ***Individual level*** |  |  |  |  |  |  |  |  |  |  |  |  |  |  |  |  |
| Education (low) |  |  |  |  |  |  |  |  |  |  |  |  |  |  |  |  |
| middle | 1.45 | *** | 1.43 | *** | 1.56 | *** | 1.42 | *** | 1.42 | *** | 1.42 | *** | 1.50 | *** | 1.42 | *** |
| High | 1.70 | *** | 1.66 | *** | 1.96 | *** | 1.65 | *** | 1.67 | *** | 1.65 | *** | 1.85 | *** | 1.64 | *** |
| Income (1st quintile) |  |  |  |  |  |  |  |  |  |  |  |  |  |  |  |  |
| 2nd quintile | 1.25 | *** | 1.25 | *** | 1.24 | *** | 1.28 | *** | 1.25 | *** | 1.24 | *** | 1.24 | *** | 1.27 | ** |
| 3rd quintile | 1.43 | *** | 1.43 | *** | 1.41 | *** | 1.46 | *** | 1.43 | *** | 1.43 | *** | 1.42 | *** | 1.44 | ** |
| 4th quintile | 1.53 | *** | 1.64 | *** | 1.51 | *** | 1.63 | *** | 1.53 | *** | 1.53 | *** | 1.51 | *** | 1.62 | *** |
| 5th quintile | 1.71 | *** | 1.73 | *** | 1.69 | *** | 1.90 | *** | 1.70 | *** | 1.72 | *** | 1.68 | *** | 1.86 | *** |
| ***Country level*** |  |  |  |  |  |  |  |  |  |  |  |  |  |  |  |  |
| Screening strategy (opportunistic) |  |  |  |  |  |  |  |  |  |  |  |  |  |  |  |  |
| Regional/rollout | 0.97 |  | 0.97 |  | 0.92 |  | 0.88 |  | 0.97 |  | 0.97 |  | 0.81 |  | 0.80 |  |
| Organized | 0.85 |  | 0.85 |  | 1.10 |  | 1.12 |  | 0.85 |  | 0.85 |  | 0.89 |  | 0.95 |  |
| ATH^a^ | 1.08 |  | 1.05 |  | 1.19 | ** | 1.19 | * | 1.05 |  | 1.05 |  | 1.06 |  | 1.06 |  |
| Decommodification^b^ | 0.95 |  | 0.95 |  | 0.88 |  | 0.88 |  | 1.00 |  | 1.02 |  | 1.18 |  | 1.13 |  |
| ***Cross-level interactions*** |  |  |  |  |  |  |  |  |  |  |  |  |  |  |  |  |
| **Education (low)*screening strategy** |  |  |  |  |  |  |  |  |  |  |  |  |  |  |  |  |
| middle*regional/rollout |  |  |  |  | 0.91 |  |  |  |  |  |  |  | 1.04 |  |  |  |
| middle*organized |  |  |  |  | 0.82 | *** |  |  |  |  |  |  | 0.96 |  |  |  |
| high*regional/rollout |  |  |  |  | 0.85 |  |  |  |  |  |  |  | 1.08 |  |  |  |
| high*organized |  |  |  |  | 0.73 | *** |  |  |  |  |  |  | 0.95 |  |  |  |
| **Income (1st quintile)*screening strategy** |  |  |  |  |  |  |  |  |  |  |  |  |  |  |  |  |
| 2nd quintile*regional/rollout |  |  |  |  |  |  | 0.98 |  |  |  |  |  |  |  | 1.07 |  |
| 2nd quintile*organized |  |  |  |  |  |  | 0.83 | ** |  |  |  |  |  |  | 0.92 |  |
| 3rd quintile*regional/rollout |  |  |  |  |  |  | 0.97 |  |  |  |  |  |  |  | 1.12 |  |
| 3rd quintile*organized |  |  |  |  |  |  | 0.82 | ** |  |  |  |  |  |  | 0.94 |  |
| 4th quintile*regional/rollout |  |  |  |  |  |  | 0.93 |  |  |  |  |  |  |  | 1.14 |  |
| 4th quintile*organized |  |  |  |  |  |  | 0.75 | *** |  |  |  |  |  |  | 0.87 | * |
| 5th quintile*regional/rollout |  |  |  |  |  |  | 0.86 |  |  |  |  |  |  |  | 1.07 |  |
| 5th quintile*organized |  |  |  |  |  |  | 0.74 | *** |  |  |  |  |  |  | 0.92 |  |
| **Education (low)*ATH\|decommodification** |  |  |  |  |  |  |  |  |  |  |  |  |  |  |  |  |
| middle*ATH\|decommodification | 0.97 | ** |  |  | 0.99 |  |  |  | 0.95 | *** |  |  | 0.93 | ** |  |  |
| high*ATH\|decommodification | 0.94 | *** |  |  | 0.97 |  |  |  | 0.91 | *** |  |  | 0.89 | *** |  |  |
| **Income (1st quintile)*ATH\|decommodification** |  |  |  |  |  |  |  |  |  |  |  |  |  |  |  |  |
| 2nd quintile*ATH\|decommodification |  |  | 1.00 |  |  |  | 1.00 |  |  |  | 0.94 | *** |  |  | 0.98 |  |
| 3rd quintile*ATH\|decommodification |  |  | 1.00 |  |  |  | 0.99 |  |  |  | 0.92 | *** |  |  | 0.99 |  |
| 4th quintile*ATH\|decommodification |  |  | 1.00 |  |  |  | 0.99 |  |  |  | 0.92 | *** |  |  | 0.99 |  |
| 5th quintile*ATH\|decommodification |  |  | 0.98 |  |  |  | 0.98 |  |  |  | 0.90 | *** |  |  | 0.98 |  |
| **Screening strategy (opportunistic)*ATH\|decommodification** | |  |  |  |  |  |  |  |  |  |  |  |  |  |  |  |
| Regional/rollout*ATH\|decommodification |  |  |  |  | 1.07 |  | 0.99 |  |  |  |  |  | 0.80 |  | 0.92 |  |
| organized*ATH\|decommodification |  |  |  |  | 0.88 |  | 0.85 |  |  |  |  |  | 0.79 |  | 0.83 |  |
| **Education (low)*screening strategy (opportunistic) *ATH\|decommodification** |  |  |  |  |  |  |  |  |  |  |  |  |  |  |  |  |
| middle*regional/rollout*ATH\|decommodification |  |  |  |  | 1.05 |  |  |  |  |  |  |  | 1.15 | ** |  |  |
| middle*organized*ATH\|decommodification |  |  |  |  | 0.94 | ** |  |  |  |  |  |  | 0.96 |  |  |  |
| high*regional/rollout*ATH\|decommodification |  |  |  |  | 1.10 |  |  |  |  |  |  |  | 1.33 | *** |  |  |
| high*organized*ATH\|decommodification |  |  |  |  | 0.91 | *** |  |  |  |  |  |  | 0.93 | * |  |  |
| **Income (1st quintile)*screening strategy (opportunistic) *ATH\|decommodification** |  |  |  |  |  |  |  |  |  |  |  |  |  |  |  |  |
| 2nd quintile*regional/rollout*ATH\|decommodification | |  |  |  |  |  | 1.13 |  |  |  |  |  |  |  | 0.99 |  |
| 2nd quintile*organized*ATH\|decommodification |  |  |  |  |  |  | 0.96 |  |  |  |  |  |  |  | 0.93 |  |
| 3rd quintile*regional/rollout*ATH\|decommodification | |  |  |  |  |  | 1.23 | ** |  |  |  |  |  |  | 0.97 |  |
| 3rd quintile*organized*ATH\|decommodification |  |  |  |  |  |  | 0.97 |  |  |  |  |  |  |  | 0.90 | ** |
| 4th quintile*regional/rollout*ATH\|decommodification | |  |  |  |  |  | 1.27 | *** |  |  |  |  |  |  | 1.00 |  |
| 4th quintile*organized*ATH\|decommodification |  |  |  |  |  |  | 0.95 | * |  |  |  |  |  |  | 0.88 | *** |
| 5th quintile*regional/rollout*ATH\|decommodification | |  |  |  |  |  | 1.25 | ** |  |  |  |  |  |  | 1.04 |  |
| 5th quintile*organized*ATH\|decommodification |  |  |  |  |  |  | 0.95 | * |  |  |  |  |  |  | 0.84 | *** |
| ^a^Access to healthcare; ^b^Welfare state decommodification | |  |  |  |  |  |  |  |  |  |  |  |  |  |  |  |
| Note: All models are adjusted for age, marital status, urbanity, self-rated health, work status, country of birth, time since last GP visit, GDP per capita and Gini | | | | | | | | | | | | | | | | |
| Note: The “ATH\|decommodification” label is used to differentiate between models on access to healthcare and models on welfare state decommodification | | | | | | | | | | | | | | | | |
| *p<0.05; **p<0.01; ***p<0.001 |  |  |  |  |  |  |  |  |  |  |  |  |  |  |  |  |

| **Appendix Table 3** Multilevel logistic regression coefficients and cross-level interaction effects in cervical cancer screening uptake by macro-level access to healthcare and decommodification, in women aged 25-64 (N=96’883) in the European Health Interview Survey | | | | | | | | |
| --- | --- | --- | --- | --- | --- | --- | --- | --- |
|  | Model 1 | | Model 2 | | Model 3 | | Model 4 | |
|  | OR | Sign | OR | Sign | OR | Sign | OR | Sign |
| ***Individual level*** |  |  |  |  |  |  |  |  |
| Education (low) |  |  |  |  |  |  |  |  |
| middle | 1.47 | *** | 1.46 | *** | 1.46 | *** | 1.45 | *** |
| High | 1.74 | *** | 1.70 | *** | 1.80 | *** | 1.68 | *** |
| Income (1st quintile) |  |  |  |  |  |  |  |  |
| 2nd quintile | 1.25 | *** | 1.24 | *** | 1.24 | *** | 1.26 | *** |
| 3rd quintile | 1.44 | *** | 1.44 | *** | 1.43 | *** | 1.45 | *** |
| 4th quintile | 1.55 | *** | 1.55 | *** | 1.54 | *** | 1.62 | *** |
| 5th quintile | 1.70 | *** | 1.72 | ** | 1.68 | *** | 1.91 | *** |
| ***Country level*** |  |  |  |  |  |  |  |  |
| Screening strategy (opportunistic) |  |  |  |  |  |  |  |  |
| Regional/rollout | 0.74 |  | 0.75 |  | 0.59 |  | 0.63 |  |
| Organized | 0.90 |  | 0.90 |  | 0.99 |  | 0.97 |  |
| ATH^a^ | 1.13 | * | 1.08 |  | 1.12 |  | 1.14 |  |
| Decommodification^b^ | 0.95 |  | 0.98 |  | 1.06 |  | 0.99 |  |
| ***Cross-level interactions*** |  |  |  |  |  |  |  |  |
| **Education (low)*screening strategy** |  |  |  |  |  |  |  |  |
| middle*regional/rollout |  |  |  |  | 1.19 | * |  |  |
| middle*organized |  |  |  |  | 0.93 |  |  |  |
| high*regional/rollout |  |  |  |  | 1.26 | ** |  |  |
| high*organized |  |  |  |  | 0.87 |  |  |  |
| **Income (1st quintile)*screening strategy** |  |  |  |  |  |  |  |  |
| 2nd quintile*regional/rollout |  |  |  |  |  |  | 1.08 |  |
| 2nd quintile*organized |  |  |  |  |  |  | 0.93 |  |
| 3rd quintile*regional/rollout |  |  |  |  |  |  | 1.13 |  |
| 3rd quintile*organized |  |  |  |  |  |  | 0.94 |  |
| 4th quintile*regional/rollout |  |  |  |  |  |  | 1.18 | * |
| 4th quintile*organized |  |  |  |  |  |  | 0.87 |  |
| 5th quintile*regional/rollout |  |  |  |  |  |  | 1.06 |  |
| 5th quintile*organized |  |  |  |  |  |  | 0.90 |  |
| **Education (low)*ATH** |  |  |  |  |  |  |  |  |
| middle*ATH | 0.97 | *** |  |  | 1.03 |  |  |  |
| high*ATH | 0.94 | *** |  |  | 1.02 |  |  |  |
| **Education (low)*decommodification** |  |  |  |  |  |  |  |  |
| middle*decommodification | 0.96 | ** |  |  | 0.91 | ** |  |  |
| high*decommodification | 0.93 | *** |  |  | 0.88 | *** |  |  |
| **Income (1st quintile)*ATH** |  |  |  |  |  |  |  |  |
| 2nd quintile*ATH |  |  | 1.02 |  |  |  | 1.01 |  |
| 3rd quintile*ATH |  |  | 1.01 |  |  |  | 0.99 |  |
| 4th quintile*ATH |  |  | 1.01 |  |  |  | 0.99 |  |
| 5th quintile*ATH |  |  | 1.00 |  |  |  | 0.97 |  |
| **Income (1st quintile)*decommodification** |  |  |  |  |  |  |  |  |
| 2nd quintile*decommodification |  |  | 0.93 | *** |  |  | 0.97 |  |
| 3rd quintile*decommodification |  |  | 0.91 | *** |  |  | 1.00 |  |
| 4th quintile*decommodification |  |  | 0.90 | *** |  |  | 1.00 |  |
| 5th quintile*decommodification |  |  | 0.90 | *** |  |  | 1.02 |  |
| **Screening strategy (opportunistic)*ATH** |  |  |  |  |  |  |  |  |
| Regional/rollout*ATH |  |  |  |  | 1.42 | * | 1.30 |  |
| organized*ATH |  |  |  |  | 0.95 |  | 0.88 |  |
| **Screening strategy (opportunistic)*decommodification** | |  |  |  |  |  |  |  |
| Regional/rollout*decommodification |  |  |  |  | 0.82 |  | 0.96 |  |
| organized*decommodification |  |  |  |  | 0.84 |  | 0.95 |  |
| **Education (low)*screening strategy (opportunistic) *ATH** |  |  |  |  |  |  |  |  |
| middle*regional/rollout*ATH |  |  |  |  | 0.88 | *** |  |  |
| middle*organized*ATH |  |  |  |  | 0.92 | ** |  |  |
| high*regional/rollout*ATH |  |  |  |  | 0.85 | *** |  |  |
| high*organized*ATH |  |  |  |  | 0.89 | *** |  |  |
| **Education (low)*screening strategy (opportunistic) *decommodification** |  |  |  |  |  |  |  |  |
| middle*regional/rollout*decommodification |  |  |  |  | 1.19 | *** |  |  |
| middle*organized*decommodification |  |  |  |  | 1.05 |  |  |  |
| high*regional/rollout*decommodification |  |  |  |  | 1.36 | *** |  |  |
| high*organized*decommodification |  |  |  |  | 1.05 |  |  |  |
| **Income (1st quintile)*screening strategy (opportunistic) *ATH** |  |  |  |  |  |  |  |  |
| 2nd quintile*regional/rollout*ATH |  |  |  |  |  |  | 1.00 |  |
| 2nd quintile*organized*ATH |  |  |  |  |  |  | 0.98 |  |
| 3rd quintile*regional/rollout*ATH |  |  |  |  |  |  | 1.00 |  |
| 3rd quintile*organized*ATH |  |  |  |  |  |  | 1.03 |  |
| 4th quintile*regional/rollout*ATH |  |  |  |  |  |  | 0.98 |  |
| 4th quintile*organized*ATH |  |  |  |  |  |  | 1.01 |  |
| 5th quintile*regional/rollout*ATH |  |  |  |  |  |  | 1.00 |  |
| 5th quintile*organized*ATH |  |  |  |  |  |  | 1.04 |  |
| **Income (1st quintile)*screening strategy (opportunistic) *decommodification** |  |  |  |  |  |  |  |  |
| 2nd quintile*regional/rollout*decommodification |  |  |  |  |  |  | 1.00 |  |
| 2nd quintile*organized*decommodification |  |  |  |  |  |  | 0.95 |  |
| 3rd quintile*regional/rollout*decommodification |  |  |  |  |  |  | 0.95 |  |
| 3rd quintile*organized*decommodification |  |  |  |  |  |  | 0.87 | * |
| 4th quintile*regional/rollout*decommodification |  |  |  |  |  |  | 0.99 |  |
| 4th quintile*organized*decommodification |  |  |  |  |  |  | 0.87 | ** |
| 5th quintile*regional/rollout*decommodification |  |  |  |  |  |  | 0.99 |  |
| 5th quintile*organized*decommodification |  |  |  |  |  |  | 0.80 | *** |
| ^a^Access to healthcare; ^b^Welfare state decommodification | |  |  |  |  |  |  |  |
| Note: All models are adjusted for age, marital status, urbanity, self-rated health, work status, country of birth, time since last GP visit, GDP per capita and Gini | | | | | | | | |
| *p<0.05; **p<0.01; ***p<0.001 |  |  |  |  |  |  |  |  |
